# Supplementary material for: Upregulation of Sulfated N-Glycans in Serum as Predictive Biomarkers for Early-Stage Breast Cancer
Source: Int J Mol Sci. 2025 May 22;26(11):4968. doi: 10.3390/ijms26114968 (PMC12154234; doi:10.3390/ijms26114968)
Supplement: Supplementary file 1 [file ijms-26-04968-s001.zip › ijms-3616862-supplementary.pdf]

Supplementary information

Upregulation of Sulfated N-Glycans in Serum as Predictive Biomarkers for Early-Stage Breast Cancer

Dereje G. Feleke[1] Bryan M. Montalban[2] Solomon T. Gizaw[3] Hiroshi Hinou*[1, 4]

[1] Laboratory of Advanced Chemical Biology, Graduate School of Life Science, Hokkaido University, N21, W11, Sapporo 001-0021, Japan

[2] Department of Physical Sciences and Mathematics, College of Arts and Sciences, University of the Philippines Manila, Padre Faura St., Manila 1000, Philippines

[3] Department of Biochemistry, College of Health Science, Addis Ababa University, P.O. Box 9086, Addis Ababa, Ethiopia

[4] Frontier Research Center for Advanced Material and Life Science, Faculty of Advanced Life Science, Hokkaido University, N21, W11, Sapporo 001-0021, Japan

*Corresponding author: email: hinou@sci.hokudai.ac.jp

Table of contents

Figure S1. Schematic workflow of the glycoblotting-based sulphoglycomics approaches for the analysis of sulfated N-glycans 2

Figure S2: Human serum calibration curve showing quantitative reproducibility of some common sulfated glycans 3

Figure S3: MALDI-TOF MS profiles of BOA-labelled IgG neutral and sulfated N-glycans from serum BC patients 4

Figure S4: Age-associated expression of serum sulfated N-glycans and age distribution of breast cancer patients 5

Table S1. Demographic characteristics of the study participants 6

Table S2: Estimated composition of sulfated N-glycans from human serum glycoproteins labeled with BOA 7

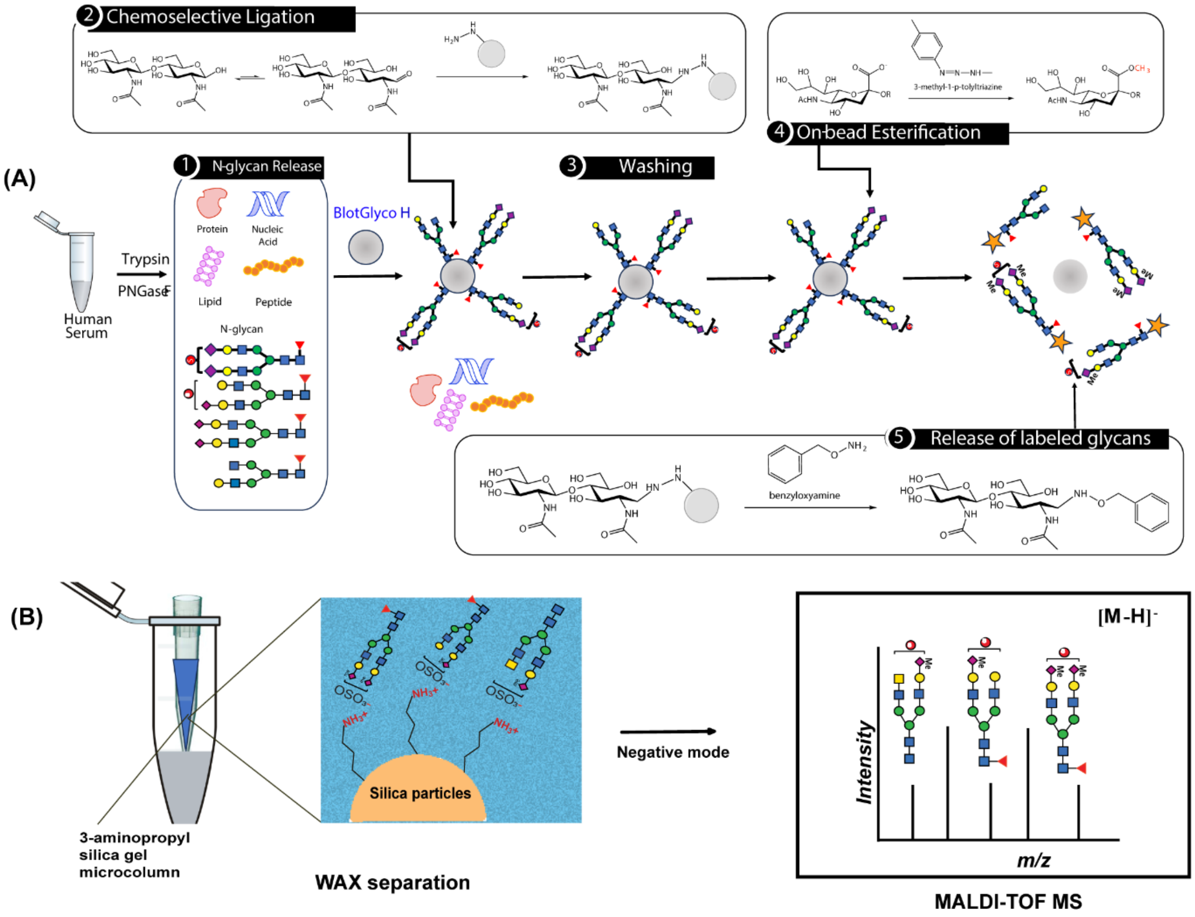

Figure S1. Schematic workflow of the glycoblotting-based sulphoglycomics approaches for the analysis of sulfated N-glycans. A) Overall workflow of glycoblotting-based MALDI-TOF M/S analysis of N-glycans derived from serum glycoproteins. 1) Enzymatic releasing of N-glycans from serum glycoproteins 2) Chemoselective ligations of N-glycans by capturing reducing sugars onto hydrazide-functionalized BlotGlyco H beads 3) Washing to remove impurities 4) On-bead methyl esterification of –COOH of terminal sialic acid residues 5) Recovery of Benzyloxyamine (BOA)-labeled glycans using trans-iminization reaction. B) Weak anion exchange (WAX) separation method using 3-aminopropyl (3-AP) silica gel microcolumn followed by MALDI-TOF M/S analysis in negative mode.

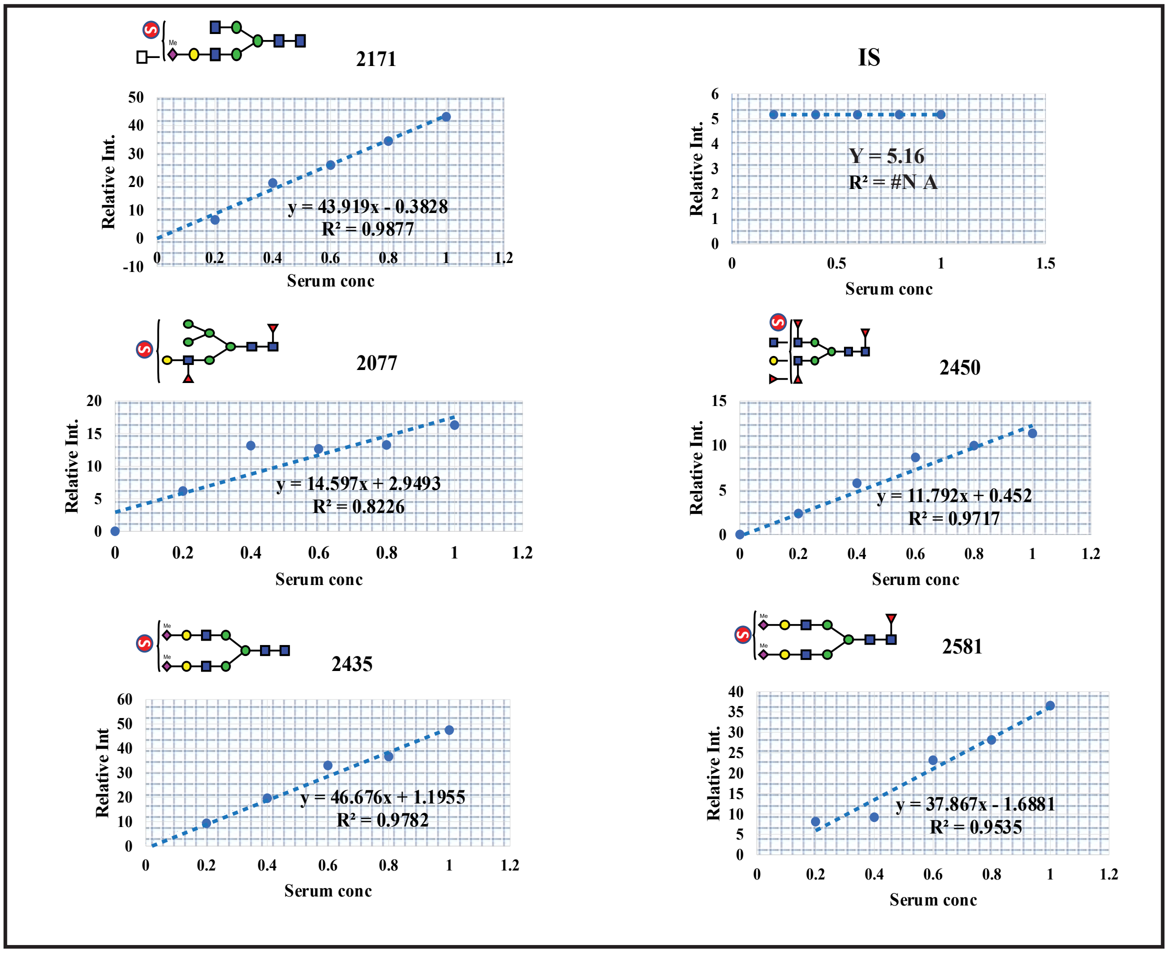

Figure S2: Human serum calibration curve showing quantitative reproducibility of some common sulfated glycans.

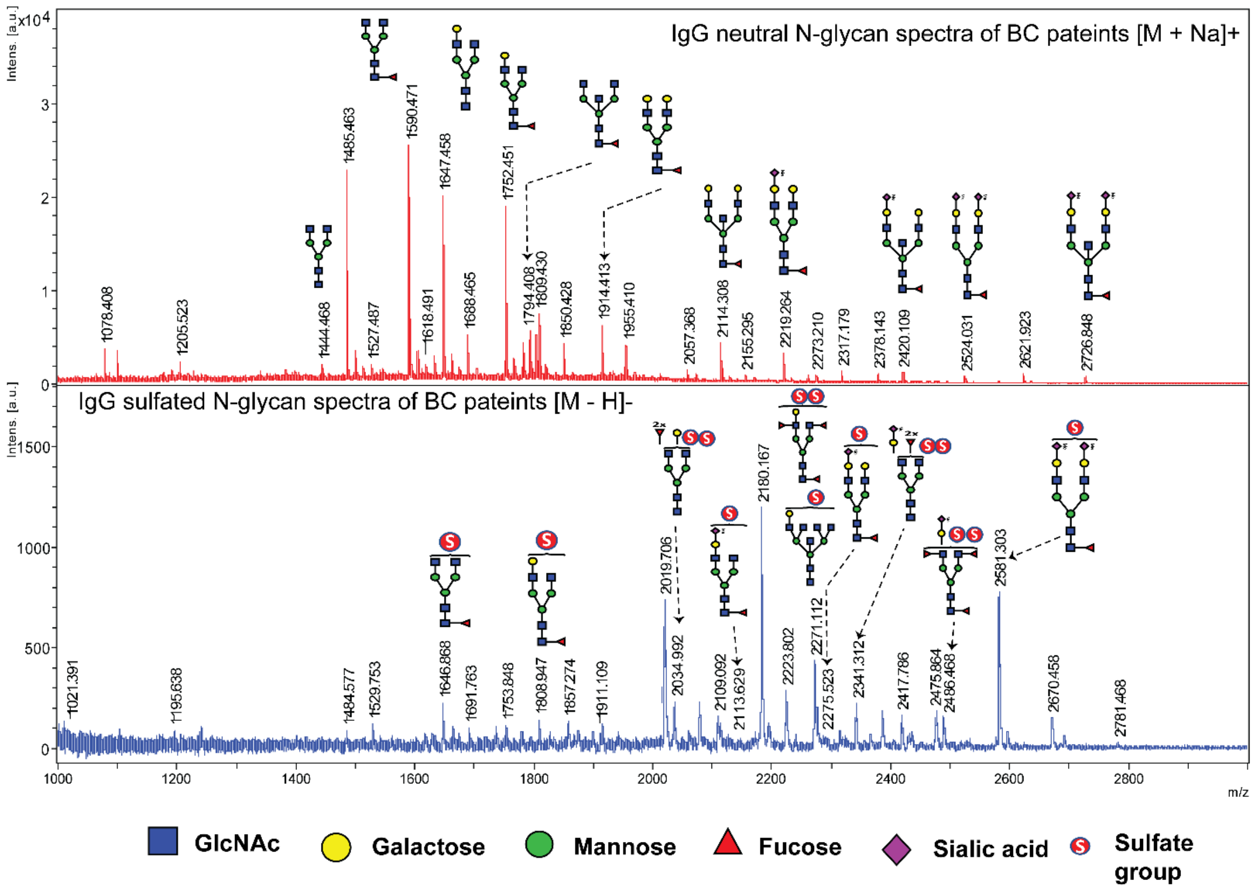

Figure S3: MALDI-TOF MS profiles of BOA-labelled IgG neutral and sulfated N-glycans from serum BC patients.

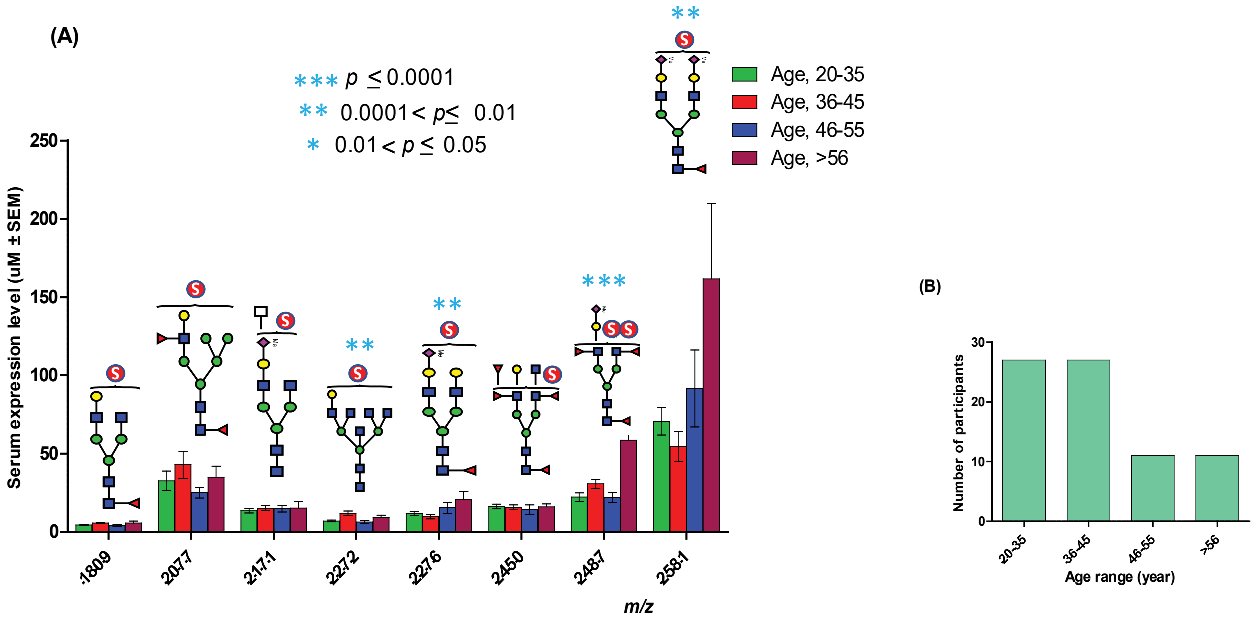

Figure S4: Age-associated expression of serum sulfated N-glycans and age distribution of breast cancer patients A) Age-associated expression patterns of serum sulfated N-glycans in breast cancer patients, **B)** Distribution of study participants across the defined age groups. The figure shows the relative expression levels of seven sulfated N-glycans identified as early-stage breast cancer biomarkers (*m/z* 1809, 2077, 2171, 2272, 2276, 2450, and 2581) across four age groups (20–35, 36–45, 46–55, and >56 years). Among these, *m/z* 2272, 2276, and 2581 exhibited statistically significant differences across age groups ($p < 0.05$, one-way ANOVA with Tukey's post hoc test). Specifically, *m/z* 2272 was highest in the 36–45 group, while *m/z* 2276 and 2581 were more elevated in patients >56 years. In contrast, *m/z* 2487, which was not identified as a BC biomarker, also showed a significant increase in the >56 group, suggesting an age-related, non-cancer-specific glycan pattern. These findings indicate that age may influence the expression of certain glycans, including some biomarkers. However, since the majority of patients in this study were under 56 years of age, and the biomarkers were still elevated in early-stage BC across age groups, it is unlikely that age alone explains their expression. This reinforces their diagnostic relevance while highlighting the value of age-adjusted models in future biomarker validation efforts.

Table S1. Demographic characteristics of the study participants

		Mean ± SD		Number of subjects per age range				
**Status**	**Number (n)**	**Age**	**BMI (kg/m2)**	**20-30**	**31-40**	**41-50**	**51-60**	**≥61**
NC	20	32.3 ± 7.54	22.33 ± 3.20	10	8	1	1	0
BC-I	17	43.0 ± 13.88	22.37 ± 2.56	4	5	3	3	2
BC-II	20	42.8 ± 11.23	21.90 ± 3.10	3	7	5	2	3
BC-III	17	39.7 ± 11.02	22.90 ± 2.66	3	9	4	0	1
BC-IV	22	40.36 ± 12.29	22.86 ± 2.87	6	7	6	2	1
**Total (N)**	**96**			**26**	**36**	**19**	**8**	**7**

Table S2: Estimated composition of sulfated N-glycans from human serum glycoproteins labeled with BOA.

**Observed Mass (m/z) [M-H]-**	**Calculated Mass (m/z) [M-H]-**	**Error (ppm)**	**Glycan Composition**	**Probable Structure [1-3]**	**GlyConnect Database Links**
1764.623	1764.589	25.06	(Hex)1 (HexNAc)1 (NeuAc)1 (Su)1 + (Man)3(GlcNAc)2	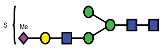	
1808.838	1808.615	120.09	(Hex)1 (HexNAc)2 (dHex)1 (Su)1 + (Man)3(GlcNAc)2	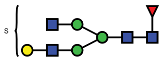	GlyConnect
1824.629	1824.61	7.79	(Hex)2 (HexNAc)2 (Su)1 + (Man)3(GlcNAc)2	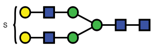	GlyConnect
1913.718	1913.646	34.6	(Hex)2 (HexNAc)1 (dHex)2 (Su)1 + (Man)3(GlcNAc)2	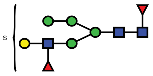	
2034.75	2034.63	56.63	(Hex)1 (HexNAc)2 (dHex)2 (Su)2 + (Man)3(GlcNAc)2	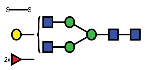	
2076.558	2075.699	410.88	(Hex)3 (HexNAc)1 (dHex)2 (Su)1 + (Man)3(GlcNAc)2	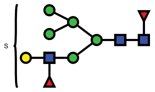	
2130.004	2129.721	137.66	(Hex)2 (HexNAc)2 (NeuAc)1 (Su)1 + (Man)3(GlcNAc)2	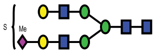	GlyConnect
2137.073	2136.66	195.57	(Hex)1 (HexNAc)1 (dHex)2 (NeuAc)1 (Su)2 + (Man)3(GlcNAc)2	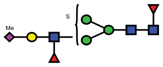	
2170.98	2170.747	112.04	(Hex)1 (HexNAc)3 (NeuAc)1 (Su)1 + (Man)3(GlcNAc)2	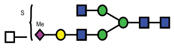	GlyConnect
2271.621	2271.795	-79.13	(Hex)1 (HexNAc)5 (Su)1 + (Man)3(GlcNAc)2	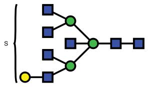	
2275.807	2275.779	16.79	(Hex)2 (HexNAc)2 (dHex)1 (NeuAc)1 (Su)1 + (Man)3(GlcNAc)2	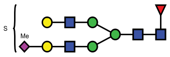	GlyConnect
2340.237	2339.741	216.31	(Hex)1 (HexNAc)2 (dHex)2 (NeuAc)1 (Su)2 + (Man)3(GlcNAc)2	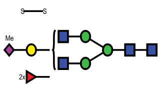	

2435.022	2434.832	88.8	(Hex)2 (HexNAc)2 (NeuAc)2 (Su)1 + (Man)3(GlcNAc)2	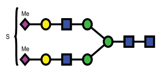	
2449.363	2449.868	-208.53	(Hex)1 (HexNAc)3 (dHex)4 (Su)1 + (Man)3(GlcNAc)2	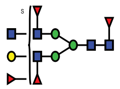	
2486.101	2485.798	125.59	(Hex)1 (HexNAc)2 (dHex)3 (NeuAc)1 (Su)2 + (Man)3(GlcNAc)2	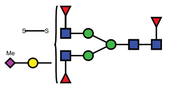	
2581.064	2580.89	77.57	(Hex)2 (HexNAc)2 (dHex)1 (NeuAc)2 (Su)1 + (Man)3(GlcNAc)2	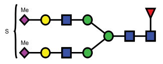	GlyConnect
3010.391	3009.984	143.91	(Hex)2 (HexNAc)3 (dHex)2 (NeuAc)2 (Su)2 + (Man)3(GlcNAc)2	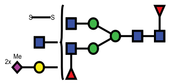	
3156.523	3156.042	160.69	(Hex)2 (HexNAc)3 (dHex)3 (NeuAc)2 (Su)2 + (Man)3(GlcNAc)2	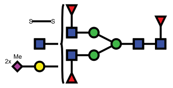	

m/z 1824 is an internal standard (IS). *m/z* values of 1764.623, 1913.718, 2130.004, and 3156.523 were not included for quantitative comparison because they were not quantitatively reproducible in the entire study samples. Monosaccharide nomenclatures are based on the SNFG: Hexose (Hex), *N*-acetyl hexoseamine (HexNAc), Mannose (Man), Fucose (Deoxyhexose), N-acetyl glucosamine (GlcNAc), Sulfate (Sulph), and *N*-acetyl neuraminic acid (NeuAc).

Reference

1. Lee J, Ha S, Kim M, Kim S-W, Yun J, Ozcan S, Hwang H, Ji IJ, Yin D, Webster MJ: **Spatial and temporal diversity of glycome expression in mammalian brain**. *Proceedings of the National Academy of Sciences* 2020, **117**(46):28743-28753.

2. Van Rooijen JJ, Kamerling JP, Vliegenthart JF: **Sulfated di-, tri-and tetraantennary N-glycans in human Tamm-Horsfall glycoprotein**. *European journal of biochemistry* 1998, **256**(2):471-487.

3. HÅRD K, VAN ZADELHOFF G, MOONEN P, KAMERLING JP, VLIEGENTHART JF: **The Asn-linked carbohydrate chains of human Tamm-Horsfall glycoprotein of one male: Novel sulfated and novel N-acetylgalactosamine-containing N-linked carbohydrate chains**. *European journal of biochemistry* 1992, **209**(3):895-915.
